# Stress management interventions for university students in low-and middle-income countries: a systematic review and meta-analysis

**DOI:** 10.3389/fdgth.2025.1603389

**Published:** 2025-09-10

**Authors:** Dilfa Juniar, Wouter van Ballegooijen, Gabrielle Kleygrewe, Anneke van Schaik, Jan Passchier, Heleen Riper

**Affiliations:** ^1^ Clinical, Neuro, and Developmental Psychology, Vrije Universiteit Amsterdam, Amsterdam, Netherlands; ^2^ Faculty of Psychology, YARSI University, Jakarta, Indonesia; ^3^ Department of Psychiatry, Amsterdam UMC, Amsterdam, Netherlands; ^4^ Amsterdam Public Health, Amsterdam UMC, Amsterdam, Netherlands; ^5^ Faculty of Psychology, Padjadjaran University, Bandung, Indonesia

**Keywords:** university student, stress management intervention, low-and-middle-income countries, meta-analysis, systematic literature review, university student mental health, university student well-being

## Abstract

**Background:**

Stress is one of major issues among university students which can lead to negative academic performance and poor quality of life. Stress-management interventions (SMIs) have been proved as being effective in helping university students cope with stress. However, most of prior studies focused on high income countries while there is still scarce evidence for low-and-middle-income countries (LMICs). The objective of the present study was to examine the effectiveness of SMIs in reducing stress level experienced by university students in LMICs.

**Methods:**

Systematic searches were carried out in PubMed, Embase, APA PsycInfo, ERIC, Web of Science, and Cochrane Central up to March 2024. Of 8180 hits, we identified 28 Randomized Control Trials to be included in the analysis. Effect size (Hedge's *g*) were calculated for stress level outcomes at post-treatment.

**Results:**

The effect size of all included studies was high and statistically significant [*g* = −0.85; 95% CI (−1.34, −0.36); *p* = .002] with high heterogeneity across studies [*I*^2^ = 92.89%; 95% CI (90.94, 94.42); *p* < 0.001]. After removing outliers, the pooled effect size was corrected to medium effect [*g* = −0.61; 95% CI (−0.75, −0.47); *p* < .001] with moderate heterogeneity [*I*^2^ = 38.9%; 95% CI (0, 62.7); *p* = .033]. Most studies had methodological limitations, including high risk of bias, small sample sizes, and the use of passive control groups (e.g., waitlist or no treatment). No significant subgroup differences were found in theoretical orientation, format of intervention, control condition, country region, and risk of bias category.

**Conclusion:**

Our results indicated that SMIs effectively reduce stress among university students in LMICs. However, the overall body of evidence is limited by concerns regarding methodological rigor, and findings should be interpreted with caution. Despite these limitations, digital formats appear to hold promising potential for further development and implementation in LMIC settings, particularly given their promising scalability and cost-efficiency.

**Systematic Review Registration:**

The study protocol was registered in the Open Science Framework. The accessible link is https://doi.org/10.17605/OSF.IO/GHSEB.

## 1 Introduction

To a certain extent, stress serves as a beneficial stimulus for human growth and development (1, 2). However, ongoing high levels of stress may lead to negative outcomes, such as psychological distress, anxiety, depression, physical illness, substance abuse, and impaired academic or work performance (3–7).

Among university students stress is a major issue as they cope with numerous stressors and transitional events in academic, social, and personal domains (8, 9). This unique combination of personal change and situational challenges creates an environment that can elevate stress levels to a problematic state, often marked by persistent feelings of worry, hopelessness, or exhaustion. Globally, studies indicate an increasing number of university students experiencing stress (5, 10, 11). Although prevalence rates vary across countries, approximately 50% of the student population experiences significant levels of stress (3, 12, 13).

In Low-and middle-income-countries (LMICs), university students often face additional stressors such as being sole providers for the family, insecurity, living in a war zone or isolated area, inadequate resources, lack of water, and poor study conditions (14). These unique challenges can exacerbate stress levels and affect the overall well-being of the university students. The inability to cope with stress has been shown to negatively impact their health behaviors manifesting as e.g., alcohol abuse, smoking, and eating disorders (15–17). Furthermore, studies have also shown that stressed university students show a decrease in their mental health status, contributing to depression (18, 19) and lower self-esteem (20). These conditions, in turn, can impair students' academic performance and social functioning, leading to significant burden at university, such as academic probation and delayed graduation, which may potentially affect their future career opportunities (7, 21, 22).

A variety of interventions developed to reduce stress level in university students utilize numerous strategies and techniques such as psychoeducation, relaxation training, cognitive behavioral therapy (CBT), social support, coping skills training, and mindfulness training (23). Stress management interventions (SMIs) have been shown to effectively reduce stress among student populations (12, 23, 24) and improve their quality of life (3). Previous meta-analyses have reported effect sizes ranging from 0.30 to 0.61 for SMIs in reducing stress levels among university students (12, 24–26).

However, most of the studies have been conducted in high income countries (HICs), and the potential benefit of SMIs for reducing stress levels are less well-established in LMICs. It is important to recognize that findings from HICs may not be generalizable to university students in LMICs due to the distinct stressors they face. Therefore, it is important to examine whether SMIs are as effective in LMICs to fill the knowledge gap. This present study is a systematic review and meta-analysis of such interventions with the aim of providing an evidence-based approach for effectiveness of SMIs in decreasing the stress levels among university students in LMICs.

## 2 Methods

The study protocol was registered in The Open Science Framework which can be retrieved via https://doi.org/10.17605/OSF.IO/GHSEB. The PRISMA 2020 guidelines for reporting the systematic review and meta-analysis were followed (27). The completed PRISMA checklist is provided in the Supplementary Material S1.

### 2.1 Search strategy

A systematic search was conducted in six bibliographic databases of PubMed, Embase, APA PsycInfo, ERIC, Web of Science, and Cochrane Central in collaboration with a librarian. The search was conducted up to 28 March 2024. Search terms included index and free term variations of university students, stress, psychotherapy, and LMIC. The full search string is provided in Supplementary Material S2. After duplicate publications were removed, two researchers (DJ and GK) independently examined titles and abstracts to remove irrelevant records and retrieved studies that potentially met inclusion criteria. A third researcher (WvB) was consulted in case of any disagreements between DJ and GK.

### 2.2 Inclusion and exclusion criteria

We included studies that meet the following inclusion criteria: 1. randomized controlled trials (RCTs) published in peer-reviewed journals, 2. studies that examined the effect of stress management interventions on stress level among university students, 3. studies conducted in low-and middle-income countries according to the World Bank data report (28), 4. studies published in English, and 5. studies that utilized a self-report stress measure to assess outcomes. Comparisons could involve any type of control condition, including no treatment, active treatment, placebo, or waitlist control. No limitations were placed on the length of the follow-up period. Studies were excluded if all included participants were recruited from clinical settings.

### 2.3 Data extraction

We extracted data regarding author information, country, and publication year. Furthermore, data related to participants characteristics (target student population, recruitment strategy, inclusion criteria) and characteristics of interventions such as intervention orientation (e.g., mindfulness, cognitive behavior therapy), intervention modalities (e.g., internet based, face-to-face), control group condition, length of program, length of follow up, and stress measurement were also extracted. To calculate the effect size, the number of participants, mean scores, and standard deviation of control and intervention conditions at post-test were extracted. Intention-to-treat data were extracted when possible. If a study reported insufficient data to calculate effect sizes, the corresponding authors were contacted to request that they provide the aggregate data. If the author did not reply, we were not to include the study in our meta-analysis.

### 2.4 Risk of bias assessment

Methodological quality of the included studies was assessed by two independent researchers (DJ and GK) using the Cochrane Collaboration Risk of Bias Assessment Tool 2 (RoB 2) (29). The following five domains were assessed: the randomisation process, deviations from the intended interventions, missing outcome data, measurement of outcome, and selection of the reported results. Each domain was scored as low, moderate/some concerns, or high. The overall risk of bias was considered as high if one or more domains were rated as high risk; as moderate or having some concerns if one or more domains were raised some concerns but none were rated as high risk; and as low if all or nearly all domains were rated as low risk, with no domain rated as high risk. Disagreements in risk of bias assessment were resolved by discussion with a third researcher (WvB).

### 2.5 Data analysis

We calculated Hedges' *g* to minimize small sample size bias using mean and standard deviation of all study groups to examine standard mean differences at post-intervention between treatment and control groups. Hedges' *g* was calculated by subtracting the stress mean score of the intervention group from the stress mean score of the control group at post-treatment, divided by the pooled standard deviation of the two groups. The pooled effect size was considered as small (0.00 ≤ Hedges'*g* < 0.3), moderate (0.3 ≤ Hedges'*g* < 0.7), and large (Hedges'*g* ≥ 0.7) (30). We applied a three-level meta-analysis to account for studies with multiple intervention arms, ensuring a more accurate estimate of the effect size (31–33).

We pooled the effect size using a random-effects model because considerable heterogeneity was expected. The *I*^2^ was calculated to assess heterogeneity which categorized as low (0%–25%), moderate (26%–50%), substantial heterogeneity (51%–75%), and considerable heterogeneity (53%–100%) (34). We also calculated the 95% confidence interval for *I*^2^ values using the method proposed by Higgins and Thompson, which adjusts for variability in study result (35).

Outliers were identified by examining the absence of overlap between the 95 percent confidence intervals of individual studies with the pooled effect size's 95 percent confidence intervals. A sensitivity analysis was performed by excluding outliers to increase the accuracy of the pooled effect size estimation. Criterion for determining statistically significant outcomes was set at *P* < .05. The R software (version 4.1.0) using the MetapsyTools package (36) and the metafor package (31) were used for computation. Publication bias was evaluated by examining the funnel plot and Egger's test for the asymmetry of the funnel plot (37). Furthermore, if asymmetry of funnel plot indicated, we proceed with estimating the number of missing studies and recalculated the effect size using the trim and fill method of Duval and Tweedie (38).

Furthermore, a subgroup analysis was conducted to assess potential moderating variables that may influence SMIs effectiveness. The variables included the region of the country, as sociocultural diversity may impact intervention effectiveness (39); different theoretical modalities, such as mindfulness-based and cognitive-behavioral approaches, as it may lead to varying levels of effectiveness in stress reduction due to differences in how these approaches target cognitive and emotional processes (40); and intervention formats, comparing face to face and online delivery as previous research suggests that different intervention formats may result in different effectiveness due to variations in personal interaction, feedback immediacy, and accessibility (41). Moreover, a subgroup analysis was performed on the impact of control conditions, such as waitlist and no treatment, on perceived effectiveness. This was done in light of previous studies (42–45) indicating that different control conditions can influence effect size estimates. Furthermore, the risk of bias category was evaluated to explore its potential effect on the effect size.

## 3 Results

### 3.1 Study characteristics

**Figure 1:**
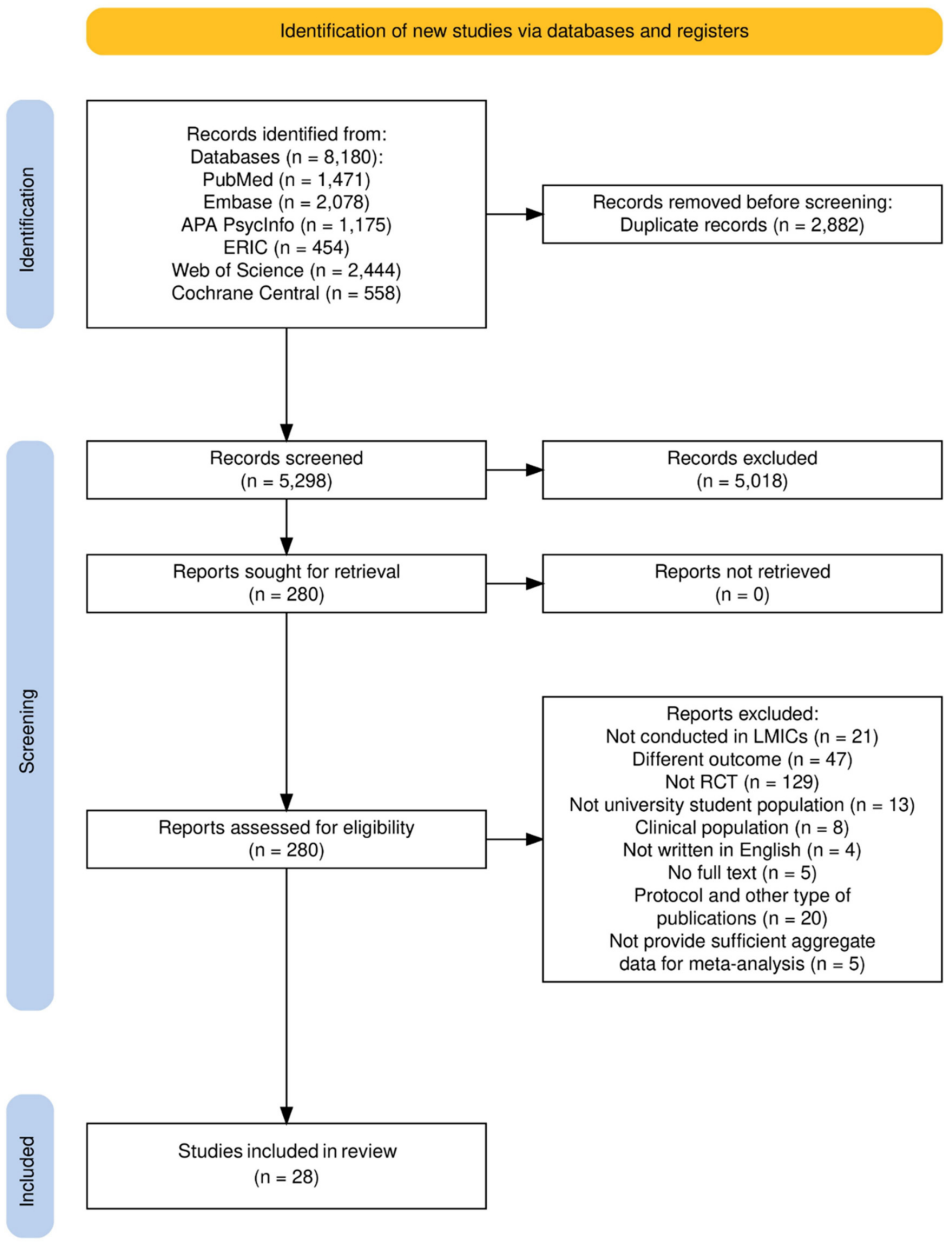
PRISMA flow diagram. Adapted with permission from “The full output plot from the PRISMA_flowdiagram() function” by Neal R. Haddaway, Matthew J. Page, Chris C. Pritchard and Luke A. McGuinness, licensed under CC BY 4.0.

The database search initially identified 8,180 studies. After removing 2,882 duplicates, 5,298 studies were screened based on titles and abstracts, resulting in the exclusion of 5,018 records due to irrelevance titles and abstracts. A total of 280 full text articles were retrieved for further assessment of eligibility. Of these, 33 met the inclusion criteria. However, six studies (46–51) lacked sufficient data for effect size calculations. Author contact efforts yielded additional data from one study (51), while two authors did not respond, one was unreachable, and two were unable to share relevant data, leading to their exclusion from the meta-analysis. In total, 28 studies with 31 comparisons were analyzed (Figure 1). These studies involved 2,995 participants, with 1,491 assigned to stress management interventions and 1,504 to control conditions. Sample sizes varied across studies, ranging from 30 to 544 randomized participants. The study selection process is detailed in the PRISMA 2020 flowchart, which was generated using the PRISMA 2020 Shiny application (52).

The included studies were conducted between 2011 and February 2024 across Asia, South America, Africa, and the Middle East. The countries represented were Brazil (*n* = 4), China (*n* = 5), Colombia (*n* = 1), Grenada (*n* = 1), India (*n* = 3), Indonesia (*n* = 1), Iran (*n* = 1), Jordan (*n* = 1), Malaysia (*n* = 1), Nigeria (*n* = 2), Thailand (*n* = 1), Tunisia (*n* = 1), Turkey (*n* = 5), and Vietnam (*n* = 1). In total, 2,553 participants were randomized, with 1,270 in intervention groups and 1,283 in control groups. Participant ages ranged from 16 to 30 years.

Sex distribution was reported in 25 studies, comprising 635 male and 1,942 female participants. Three studies exclusively recruited female students due to the research objectives (53), the institution in which the research was conducted is female-specific (54), or a low proportion of male students (55).

The participant population consisted of undergraduate and graduate students, with the majority being freshmen and sophomores in medical and nursing faculties. Only two studies included graduate students as participants (56, 57). Of the 28 studies reviewed, four did not specify any inclusion or exclusion criteria. These studies only required participants to provide consent, without detailing exclusion criteria. The remaining studies outlined various inclusion and exclusion criteria, which ranged from age restrictions (e.g., a minimum age of 18) to specific baseline measurements (e.g., scoring above 14 on the stress subscale of the DASS). Other criteria included having no prior experience with a particular therapy or intervention and not having a diagnosed mental disorder. Additionally, not all studies explicitly stated whether participation was voluntary.

The integration of interventions within university settings varied across studies. Four interventions were incorporated into mandatory or elective courses, 15 were delivered as independent programs, and nine did not specify how they were integrated into the academic curriculum. Only three studies reported offering incentives for participation. Recruitment strategies were primarily campus-based, including course enrollment and classroom announcements, while others utilized online methods such as email, social media platforms, and promotional videos. Incentives were reported in three studies, provided in the form of monetary compensation or course credit.

Stress management approaches were primarily dominated by mindfulness-based interventions (*n* = 13), with three studies implementing brief programs consisting of three to four sessions. Other approaches included psychoeducation (*n* = 4) and cognitive-behavioral therapy (*n* = 5), which encompassed cognitive-behavioral techniques, critical thinking, problem-solving training, and positive psychology interventions. Additionally, mind-body-based interventions (*n* = 7), such as yoga and physical exercise, were also utilized to manage stress among university students in LMICs (54, 55, 58–62).

Most interventions were delivered face-to-face (*n* = 21). The majority of these were conducted individually (*n* = 17), typically involving direct interaction with a facilitator. The remaining four were delivered in a group setting (53, 59, 63, 64). In contrast, online formats were less common (*n* = 7). Among the online interventions, five followed an individual (non-group) format including three unguided programs (65–67), one that was guided (68), and one that was partially guided or with limited support provided only during the first one or two sessions (69). Two studies implemented a group-based online guided format (56, 70). Overall, most intervention (*n* = 25) were facilitated by trained professionals, while three studies employed unguided self-help format (65–67).

The duration of interventions ranged from three days to 12 weeks, with the number of sessions varying from three to 84. In the longest intervention, participants engaged in daily sessions over 12 weeks (54). Most studies implemented weekly sessions (*n* = 17), with each session lasting between 25 and 120 min. In some studies (*n* = 4), participants were required to complete the intervention daily, with session durations ranging from 15 to 35 min. Other studies (*n* = 7) conduct sessions twice a week, with each session lasting between 60 and 120 min. In terms of total session count, ten studies offered interventions with up to seven sessions, 13 studies ranged from eight to 20 sessions, and five studies exceeded 20 sessions.

The included studies utilized various control conditions. The most common was a no-treatment control group (*n* = 13), in which participants did not receive any intervention and only completed pre-test and post-test assessments. A waiting list control group was used in 10 studies, allowing participants to access the intervention after the post-test. One study employed an active control condition (68), which involved theoretical courses on stress management combined with counseling. Additionally, four studies used attention control conditions, incorporating activities such as music-based relaxation, courses on organizational aspects of the school or department, and health-related audio programs (57–59, 64).

Stress outcomes were assessed using various validated measures, including the Perceived Stress Scale (PSS; *n* = 14) and the Depression, Anxiety, and Stress Scale (DASS; *n* = 10). Additional validated stress measures, such as the Global Assessment of Recent Stress Scale and the Nursing Education Stress Scale, were used in four studies.

Follow-up assessments were conducted in 15 studies, ranging from one to six months post-intervention. Five studies used a one-month follow-up (59, 63, 67, 69, 71), two studies implemented a two-month follow-up (51, 72), five studies used a three-month follow-up (54, 66–75), and three studies included a six-month follow-up (68, 70, 75).

Of the 28 studies, only 6 explicitly stated that the intervention had been culturally adapted (e.g., through language tailoring, incorporation of cultural values, or contextual modifications) (53, 56, 72, 74–76). Two studies mentioned translation only, without further cultural adjustments (51, 77). Three studies explicitly reported that no cultural adaptation was conducted, typically because the interventions were mind-body based and considered culturally neutral (54, 58, 62). The majority of studies (*n* = 17) provided no information regarding whether any form of cultural or contextual detailed adaptation had been implemented.

Dropout rates varied widely, ranging from 0% (54–56, 58, 64–66, 68–70, 72, 73, 78, 79) to 63.79% (74). See Table 1 for study and intervention characteristics.

**Table 1:** Study characteristics.

Author (year), country	Intervention	Control condition	N Rando-mized	Participants	Inclusion criteria	Exclusion criteria	Recruitment	Format	Intervention provider	Session's length	Program integration with academic course	Follow-up (month)	Outcome	Age range (years)	Cultural Adaptation	Drop-out (%)
Alhawatmeh et al. (77), Jordan	Mindfulness	NT	112	Undergraduate nursing students	Aged 18 years or more; enrolled in a clinical subject	Participating in any type of relaxation techniques; taking psycho-active drugs	In campus using convenience sampling	Ftf; Individual; Guided	Professional	5 sessions in 5 weeks (30 min/session)	NI	None	PSS	NI	Translation	3.57
An et al. (51), Vietnam	Mindfulness	NT	49	College students	NI	DASS score: Depressio*n* > 21, Anxiety > 15, Stress > 26	NI	Ftf; Individual; Guided	Professional	8 sessions in 8 weeks (90 min/session)	Standalone	2	DASS	18–22	Translation	6.12
Bani Ahmad et al. (65), Turkey	Psycho-education	WL	60	International nursing students	Enrolled in spring semester 2020; provide consent; speaks and reads English	NI	In campus, volunteer	Online (asynchronous class); Individual; Unguided	NA	7 sessions in 6 weeks (25−60 min/session)	NI	None	PSS	17–20	NI	0
Chawla et al. (58), India	Whole-body vibrating	Exercise group without vibration	30	College students	DASS score: Depression > 10; Anxiety > 8; Stress > 15	Involved in routine exercise; Taking medication for mental health; Having lower limb prosthesis; History of any orthopedic injury, neurological disorders; Having a very high score on the DASS	NI	Ftf; Individual; Guided	Professional	8 sessions in 4 weeks (time duration/session: NI)	NI	None	DASS	NI	No	0
Cheng & Wong (59), China	Guided imagery with music	Relaxation incorporating music listening	64	Undergraduate students	No history of diagnosed psychiatric disease and/or current acute mental problems, not having a dislike of music, not having previous experience with music therapy involving mental imaging	NI	In campus, by invitation	Ftf; Group; Guided	Professional	6 sessions in 6 weeks (120 min/session)	Standalone	1	PSS	NI	NI	25.56
Damião Neto et al. (64), Brazil	Mindfulness	Course containing organizational aspects of the medical school	141	First year medical students	At least 18 years old; signed the consent form	Did not fill out all questionnaires; Withdrew from medical school; Participants who were not present when data was collected	In campus by course enrollment	Ftf; Group; Guided	Professional	6 sessions in 6 weeks (120 min/session)	Integrated as compul-sory course	None	DASS	NI	NI	0
Gallo et al. (76), Brazil	Mindfulness	WL	136	University students	Participate in mindfulness sessions at universities where the study was conducted	NI	Social media and an initial presentation about research procedures and objectives.	Ftf; Individual; Guided	Professional	8 sessions in 8 weeks (time duration/session: NI)	NI	None	PSS	18–41	Yes	44.12
Gopal et al. (54), India	Yoga	NT	60	First year medical students	Did not suffer from any acute or chronic physical illness	NI	In campus, volunteer	Ftf; Individual; Guided	Professional	Every day for 12 weeks (35 min/session)	Standalone	None	GARS	17–20	No	0
Günaydin (63), Turkey	Psycho-education	NT	59	Second year nursing students	No mental diagnosis; no use of psychiatric medications; having elevated DASS score	NI	In faculty using lottery method	Ftf; Group; Guided	Professional	7 sessions in 7 weeks (45–60 min/session)	Standalone	1	DASS	18–19	NI	35.59
Igbokwe et al. (72), Nigeria	REBT	NT	116	Undergraduate English education students	Having high PSS score; not involved in any stress intervention program; agree to complete the program; have a functional email and WhatsApp	NI	In campus, volunteer	Ftf; Individual; Guided	Professional	20 sessions in 10 weeks (75 min/session)	NI	2	PSS	19–23	Yes	0
Karaca & Sisman (78), Turkey	Mindfulness	NT	114	Second year nursing students	Willing to take the Coping with Stress course; voluntary participation	NI	In campus, volunteer	Ftf; Individual; Guided	Professional	24 sessions in 12 weeks (90–95 min/session)	Integrated as elective course	3	NESS	NI	NI	0
Komariah et al. (69), Indonesia	Mindfulness	WL	61	University students	Willing to participate in the program; 18 years or older	Severe mental health disorders	Open recruitment and flyer posted on social media	Online (Zoom); Individual; Half guided	Professional	Every day for 4 weeks (15 min/session)	Standalone	1	DASS	NI	NI	0
Krifa et al. (66), Tunisia	Positive psychology	WL	366	First to third year health care students	Being fluent in French; Aged 18–30 years; Having an email address; Have access to the internet at home	NI	NI	Online (Web-based); Individual; Unguided	NA	8 sessions in 8 weeks (45 min/session)	Standalone	3	DASS	18–30	NI	11.48
Okide et al. (73), Nigeria	Critical Thinking	WL	44	Undergraduate of adult education and extramural studies	High perceived stress based on PSS score; Consent to participate.	NI	In campus via the study program	Ftf; Individual; Guided	Professional	12 sessions in 6 weeks (120 min/session)	NI	3	PSS	19–30	NI	0
Pan & Zhuang (74), China	Adventure-based cognitive-behavioral	WL	544	Undergraduate university students	Chinese nationality; General Health Questionnaire-12 (GHQ-12) score of 2–10 at pretest	Having one or more psychotic disorders or experiencing severe depression with suicidal attempts/ideation in the 3 months before recruitment	In campus, by invitation via the university course enrollment system	Ftf; Individual; Guided	Professional	13 sessions in 13 weeks (120 min/session)	Integrated as compul-sory course	3	PSS	18–30	Yes	63.79
Phang et al. (75) , Malaysia	Mindfulness	NT	75	First to third year medical students	Provide consent	Did not attend 80% of all sessions; Not spent 3–5 min/day to practice	Advertised as extra-curricular activities via emails, Facebook, and blog	Ftf; Individual; Guided	Professional	5 sessions in 5 weeks (120 min/session)	Standalone	6	PSS	NI	Yes	6.67
Ratanasiripong et al. (55), Thailand	a. Mindfulness b. Biofeedback	NT	89	Second year nursing students	Provide consent	NI	In campus, volunteer	Ftf; Individual; Half guided	Professional	3 times a day for 4 weeks (time duration/session: NI)	NI	None	PSS	18–21	NI	0
Rentala et al. (53), India	Psycho-education	NT	230	College student	DASS score: Stress > 14; provide consent	NI	In campus, volunteer	Ftf; Group; Guided	Professional	8 sessions in 4 weeks (90–120 min/session)	Standalone	3	DASS	16–19	Yes	9.13
Senocak & Demirkiran (71), Turkey	Problem-solving training	WL	72	Second year nursing students	Attending surgical nursing course for the first time	Repeating the second year of nursing school	Course participation	Ftf; Individual; Guided	Professional	8 sessions in 7 weeks (55–150 min/session)	Integrated as compul-sory course	1	PSS	NI	NI	1.4
Silva et al. (67), Brazil	Brief- mindfulness	WL	48	University students	Aged 18–35, reside in Brazil; have access to cell phone; willingness to access the intervention app; DASS score > 0	Practicing mindfulness, using psychotropic medication, undergoing psychological treatment, or having diagnosis of serious psychiatric disorder	Promotional videos via media platforms including Facebook, WhatsApp, and YouTube	Online (Applica-tion); Individual; Unguided	NA	4 sessions in 4 weeks (30 min/video)	Standalone	1	DASS	18–34	NI	43.75
Sousa et al. (57), Brazil	Brief-mindfulness	Coloring pictures and listening to audio on health-related topics	43	Graduate and undergraduate students	Absence of psychiatric disorders, psychotropic or anti-inflammatory prescriptions; Had experience with meditation or yoga	NI	Online recruitment	Ftf; Individual; Guided	Professional	3 sessions in 3 consecutive days (30 min/session)	Standalone	None	PSS	18–30	NI	6.98
Tahsini et al. (60), Iran	BFRT	NT	30	Second year university student preparing for final examination	Having higher cut-point scores in DASS; Second year students taking 18–20 credits	History of anxiolytic, antidepressant or other psychiatric medication; History of past and current psychotherapy and biofeedback or relaxation training; smokers; alcohol users	In campus, volunteer	Ftf; Individual; Guided	Professional	8 sessions in 4 weeks (90 min/session)	NI	None	DASS	19–23	NI	3.33
Torres Lancheros et al. (56)olumbia	Brief manfulness and self-compassion	WL	35	Undergraduate and graduate students	Over 18, presenting clinically significant indicators of emotional symptoms based on the DASS score	Receiving psychological treatment, having previously received clinical diagnosis, presenting indicators of suicidal ideation	Course enrollment	Online (Google meet); Group; Guided	Professional	4 sessions in 4 weeks (120 min/session)	Standalone	None	DASS	NI	Yes	0
Waechter et al. (61), Grenada	Wellness program (Yoga, Walking, and Mindfulness)	NT	101	First year medical students	Enrolled in Basic Science Studies; Willing to attend the intervention (if assigned); willing to complete a weekly log; Willing to complete pre- and post- assessment	NI	Email, website	Ftf; Individual; Guided	Professional	24 sessions in 12 weeks (60 min/session)	Standalone	None	PSS	24–27	NI	30.69
Wang et al. (68), China	Psycho-education	Ftf theoretical courses on stress management and counselling	114	Undergraduate nursing students	Aged minimum 18 years or more; had taken clinical practice courses; provide written informed consent	Participate in another research program; Previous exposure to psychological intervention programs	In campus by research assistant's assessment	Online (Mobile phone-based); Individual; Guided	Professional	8 sessions in 8 weeks (90 min/session)	NI	6	SNSS	22–24	NI	0
Yang et al. (70), China	Mindfulness	WL	66	Undergraduate university students	> 18 years old; diagnosis of social media addiction; no related treatment experience; willingness to participate	Severe mental health disorder diagnosis	Online propaganda	Online (Web-based/Conference software); Group; Guided	Professional	8 sessions in 8 weeks (50–60 min/session)	Standalone	6	PSS	17–24	NI	0
Yildrim & Akman (62), Turkey	Acupressure	NT	98	First year nursing students	Aged minimum 18 years or more; Did not have communication problem; Stress severity >=4 (VAS); Had no prior knowledge of acupressure; Had no prior experience of clinical practice	NI	NI	Ftf; Individual; Guided	Professional	3 sessions in 3 weeks (30 min/session)	Standalone	None	VAS	18–25	No	7.14
Ying et al. (79), China	Mindfulness	NT	38	First year college students	Provide consent	NI	In campus, by presenting the study to freshmen	Ftf; Individual; Guided	Professional	8 sessions in 8 weeks (time duration/session: NI)	Standalone	None	PSS	20–30	NI	0

NI, no information; NA, not applicable; REBT, Rational Emotive Behavior Therapy; BFRT, biofeedback-aided relaxation training; NT, no treatment (assessment only); WL, waitlist; Ftf, face to face; PSS, Perceived Stress Scale; VAS, Visual Analog Scale; DASS, Depression; Anxiety, and Stress Scale, GARS, Global Assessment of Recent Stress Scale; NESS, Nursing Education Stress Scale; SNSS, Stressor in Nursing Students Scale.

### 3.2 Risk of bias

**Figure 2:**
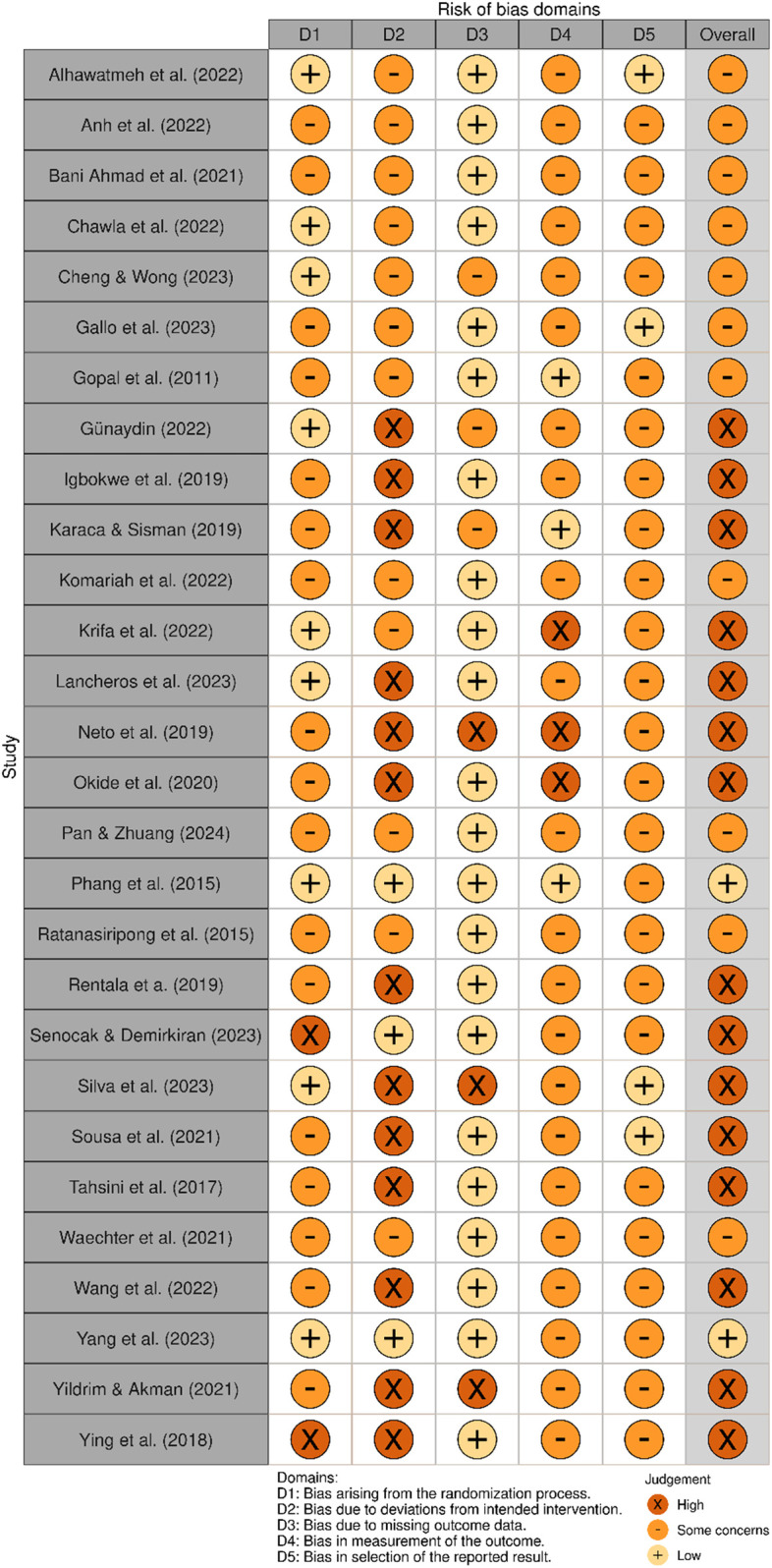
Risk of bias summary for each included study.

**Figure 3:**
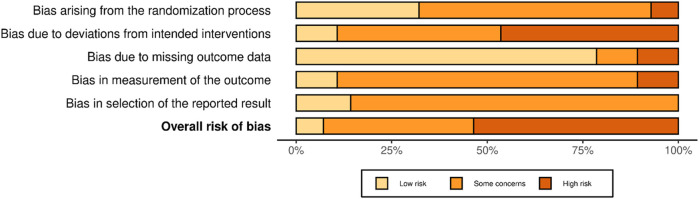
Risk of bias summary for domains.

The visualization of the risk of bias analysis is presented in Figures 2, 3. These figures were generated using the robvis tool (https://mcguinlu.shinyapps.io/robvis/) (80). Overall, two studies were classified as having a low risk of bias, 12 studies showed some concerns, and 14 studies were identified as having a high risk of bias.

The included studies reported using computer-generated randomization programs for the randomization process. However, information on allocation sequence concealment was rarely provided. This lack of clarity resulted in the majority of studies (*n* = 17) being categorized as having some concerns in this domain. Additionally, 14 studies showed some concerns, while 11 studies were rated as having a high risk of bias due to deviations from the intended intervention. This was mainly due to the lack of assessment or reporting on potential contamination between trial arms. In such cases, control participants may have inadvertently encountered key elements of the intervention through external sources, potentially influencing the findings.

Furthermore, only five studies explicitly reported using intention-to-treat analysis to estimate the intervention's effect appropriately. The risk of bias due to missing outcome data was the domain where most studies met the criteria for low bias (*n* = 22). However, bias in outcome measurement raised concerns in most studies (*n* = 19), primarily because self-reported assessments may have led participants, acting as outcome assessors, to be aware of the intervention they received, potentially influencing the outcome assessment.

Most included studies (*n* = 24) were categorized as having some concerns regarding bias in the selection of reported results, as only 10 studies had a pre-registered protocol. Additionally, four of these studies did not provide a link or sufficient information to access the protocol.

### 3.3 Primary outcome

**Figure 4:**
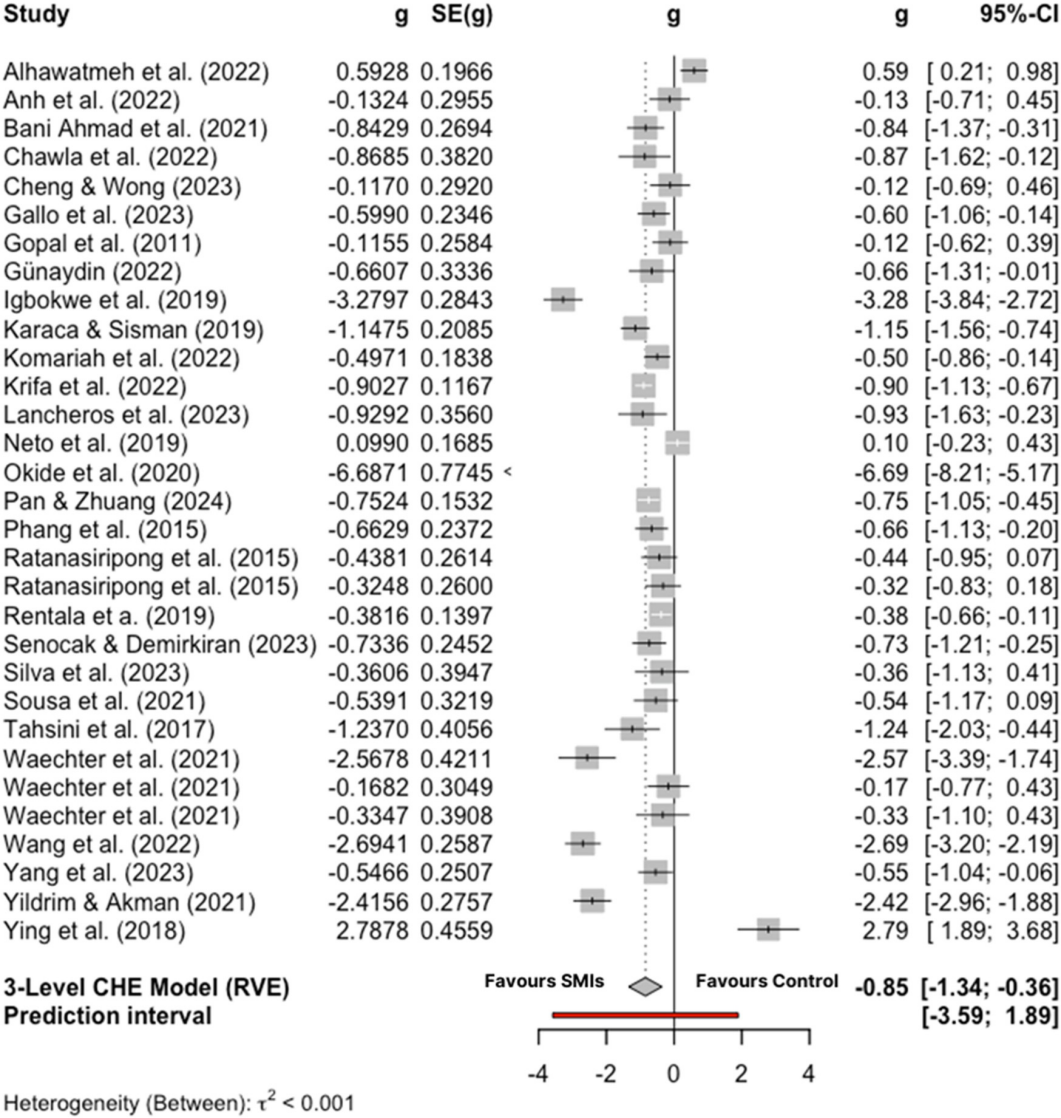
Forest plot of included studies.

**Figure 5:**
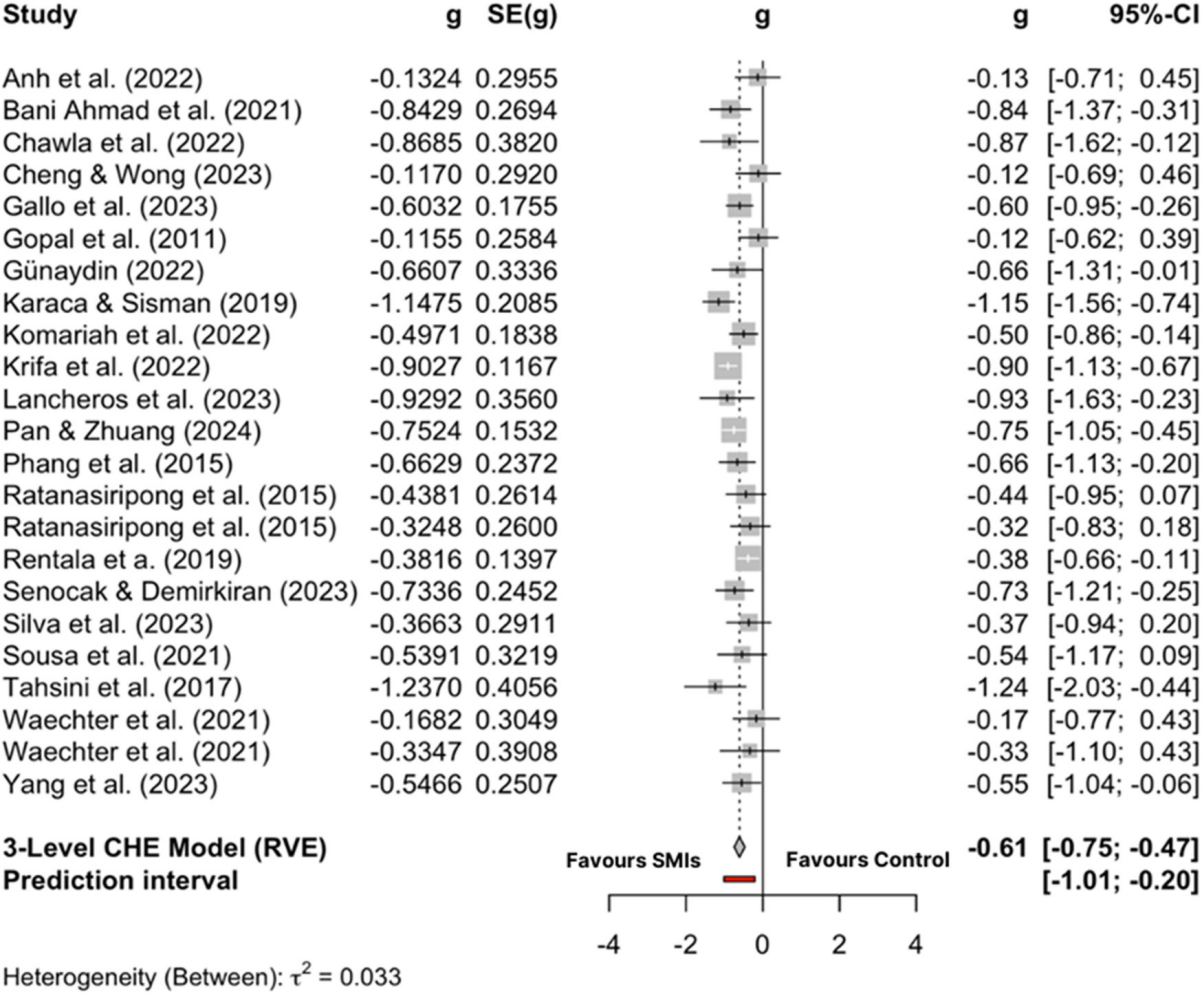
Forest plot of included studies excluding outliers.

The overall effect size of stress management interventions in comparison to control conditions at post-test was large and significant [*g* = −0.85; 95% CI (−1.34, −0.36); *p* = .002] with considerable heterogeneity across studies [*I*^2^ = 92.89%; 95% CI (90.94, 94.42%); *p* < .001]. After inspection of the forest plot (Figure 4), eight comparisons were found to be outliers (61, 62, 64, 68, 72, 73, 77, 79). After removing the outliers, the pooled effect size was corrected to medium effect size [*g* = −0.61; 95% CI (−0.75, −0.47); *p* < .001] with moderate heterogeneity [*I*^2^ = 37.9%; 95% CI (0, 62.39); *p* = .033] (see Figure 5 for forest plot).

**Figure 6:**
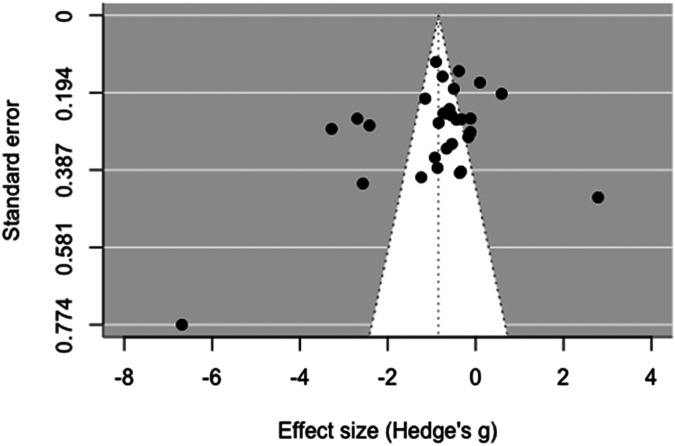
Funnel plot of all included studies.

There was publication bias indicated based on the funnel plot examination of all included studies (Figure 6). The Egger's test yielded significant results suggesting funnel plot asymmetry (intercept: 1.21; *t* = 2.02; *p* = 0.040). However, Duval and Tweedie's trim and fill procedure did not identify any missing studies, suggesting that publication bias may not be a significant concern. Consequently, the adjusted effect size remained unchanged [*g* = −0.85; 95% CI (−1.34, −0.36); *p* = .002].

### 3.4 Subgroup analysis

We conducted subgroup analyses to explore potential moderators, including country region, intervention type, format of the intervention, control condition, and risk of bias category. We found no statistically significant differences for any of the variables examined (Table 2).

**Table 2:** Subgroup analysis of included studies (*N* comparisons = 31).

Subgroup variables	Number of comparisons	Hedges' *g* (95% CI)	*I*^2^ (95% CI)	*p*
Region of country				
Middle East	7	−0.91 [−1.74; −0.07]	93.20 [88.50; 96.00]	0.290
Southeast Asia	5	−0.44 [−0.66; −0.22]	0 [0; 79.20]	
South Asia	3	−0.37 [−0.96; 0.21]	25.40 [0; 92.20]	
East Asia	5	−0.29 [−2.72; 2.14]	96.70 [94.50; 98.00]	
Latin America	8	−0.64 [−1.30; 0.03]	82.00 [65.80; 90.60]	
Africa	3	−3.55 [−10.73; 3.62]	98.20 [96.60; 99.00]	
Theoretical orientation				
Mindfulness-based	14	−0.36 [−1.00; 0.28]	89.8 [84.70; 93.20]	0.240
Psychoeducation-based	4	−1.14 [−2.82; 0.53]	95.2 [90.60; 97.50]	
Cognitive behavioral	5	−2.40 [−5.55; 0.76]	96.70 [94.50; 98.00]	
Mind-body	8	−0.69 [−1.37; −0.01]	87.40 [77.40; 93.00]	
Format of the intervention				
Face to face	24	−0.82 [−1.49; −0.15]	93.50 [91.40; 95.00]	0.730
Online	7	−0.97 [−1.71; −0.24]	89.40 [80.70; 94.20]	
Control condition				
Waitlist	10	−1.27 [−2.53; −0.01]	85.70 [75.40; 91.60]	0.600
No treatment	16	−0.62 [−1.34; 0.11]	94.40 [92.20; 95.90]	
Other	5	−0.82 [−2.21; 0.57]	95.30 [91.70; 97.40]	
Risk of Bias				
Low & Some concerns	16	−0.49 [−0.81; −0.16]	77.70 [64.20; 86.10]	0.160
High	15	−1.24 [−2.33; −0.14]	95.70 [94.20; 96.80]	

## 4 Discussion

We conducted a meta-analysis on the effectiveness of SMIs in reducing stress among university students in LMICs. A total of 28 studies with 31 comparisons were included in the meta-analysis. We found a significant high effect size with high heterogeneity across all included studies. After conducting a sensitivity analysis by removing outliers, we discovered a moderate and significant effect size of SMIs compared to the control condition with moderate heterogeneity across studies.

Our findings align with previous meta-analyses that have demonstrated the effectiveness of stress management interventions (SMIs) in reducing stress among college students (23, 26). Yusufov et al. (23) reported a moderate effect size [*d* = 0.44, 95% CI (0.24, 0.64), *p* < .01] among undergraduate and graduate students, while Amanvermez et al. (23) found a moderate pooled effect size [*g* = 0.56, 95% CI (0.44, 0.68), *p* < .001] in studies involving unselected college students. However, the majority of studies included in these meta-analyses were conducted in HICs, and no separate analysis was performed for HICs and LMICs, limiting the generalizability of their findings to students in LMICs.

Therapeutical orientation used among SMIs in LMICs varied, with mindfulness-based interventions being the most commonly used (*n* = 14). This aligns with global trends, as mindfulness-based interventions have been increasingly adopted in both HICs and LMICs due to their effectiveness in reducing stress, anxiety, and depression (81). Mindfulness-based SMIs' effectiveness has also been demonstrated specifically among university students (82–84). The preference for mindfulness-based SMIs in LMICs may be attributed to their cultural relevance and alignment with regional values, beliefs, and practices. Many LMICs have long-standing contemplative traditions rooted in Buddhist, Hindu, and Islamic philosophies, which emphasize self-awareness, acceptance, and emotional regulation—all core principles of modern mindfulness interventions (85). Additionally, mindfulness-based SMIs are often embedded within preventive mental health approaches, which are generally perceived as less stigmatizing than treatment-focus intervention in LMICs, where mental health stigma remains a significant barrier to care (86).

Mind-body-based SMIs such as physical exercise and yoga were the second most utilized approach (*n* = 8). These interventions may provide a non-stigmatizing and widely accepted means of reducing stress particularly in low resources setting. Effectiveness of mind-body based SMIs in reducing stress, anxiety, and depression among adult and college students is supported by previous RCTs (87, 88). Furthermore, a cross-sectional study among university students in LMICs found that physical activity was associated with improved stress regulation and well-being (89). Similarly, systematic reviews have reported that mind-body based intervention, including yoga and structured movement therapies, contribute to stress reduction (90, 91). Mind-body SMIs may be preferred in LMICs due to their social acceptability, and ease of implementation as physical exercise and yoga can be integrated seamlessly into daily life. Beside stand-alone intervention, mind-body interventions may serve as a complementary component within more intensive SMIs programs, enhancing overall effectiveness by addressing physical well-being alongside other psychological strategies (91).

Internet-based interventions have expanded in LMICs, particularly during and after the COVID-19 pandemic. Our findings indicate that both face-to-face and online formats are effective in delivering SMIs to university students. However, previous meta-analyses suggest that internet-based interventions, particularly those delivered without support, tend to yield smaller effects in reducing stress compared to face-to-face interventions (92–94). This may be attributed to lower engagement and higher dropout rates in unguided internet-based interventions. The guided format that incorporates professional or facilitator support has demonstrated better adherence and stronger outcomes (95, 96). Despite this, digital interventions remain a viable alternative for early intervention, particularly in low-resource settings, where access to traditional mental health services is often limited.

The predominance of face-to-face SMIs in the present study suggests that in-person formats remain highly valued in LMICs, while digital interventions are emerging but remain underrepresented in RCTs. However, with increasing technological accessibility, the growth of digital mental health fields, and the “digital native” characteristics of university students, online SMIs have significant potential for expansion. Studies show that university students perceive internet-based interventions positively and report significant benefits (97, 98). These interventions also offer scalability and accessibility, enabling them to reach a diverse student population in LMICs. Compared to face-to-face interventions, digital programs eliminate geographical barriers, allow users to engage at their convenience, and can be disseminated to large populations without a proportional increase in resources, making them a potentially cost-effective solution for student mental health in LMICs (99, 100). Recent advances in artificial intelligence (AI) also present promising future directions, particularly through virtual therapists and chatbots that may offer more conversational and human-like interactions, further enhancing the relatability and accessibility of digital interventions in these settings (101).

Despite the growing interest in digital interventions, existing digital programs in LMICs primarily focus on clinical conditions such as depression, anxiety, post-traumatic stress disorder, and substance misuse (102), with limited emphasis on subclinical and preventive applications. Concurrently, a wide range of digital tools such as apps and wearable-supported platforms that promote exercise, yoga, and mindfulness are now available to support self-care and general well-being. These tools represent important developments in the broader digital mental health landscape. However, their usage and effectiveness among university students in LMICs remain underexamined. Expanding culturally adapted, low-intensity interventions particularly unguided and group-based formats could help bridge existing gaps in student mental health care. Given the barriers to access individualized psychological support in LMICs, integrating low-intensity, scalable interventions within university settings may improve accessibility to mental health services.

Among the included studies, most SMIs (*n* = 25) were delivered by trained professionals, with no studies utilizing lay providers. In LMICs, lay personnel have been increasingly recognized as a viable resource for expanding mental health services, particularly in settings with limited access to professional mental health care (103). Given the importance of peer influence during university years, integrating peer counselors into structured, low-intensity interventions may be promising in university settings. While concerns have been raised about the quality and consistency of care delivered by non-professionals, evidence suggests that, when supported by proper training, supervision, and clear intervention guidelines, lay providers can deliver mental health intervention effectively and safely (104). Incorporating trained peer counselors into university-based programs may thus enhance feasibility, accessibility, and engagement in university-based mental health programs without compromising intervention quality (105).

While non-group formats dominated in the included studies, some interventions adopted group-based approaches, which may provide a cost-effective alternative for delivering SMIs in low-resources university settings. Although evidence specifically among university students in LMICs remains limited, studies from other youth population suggest promising outcomes. RCTs in Kenya and China have demonstrated the effectiveness and cost-effectiveness of group-and school-based interventions for adolescents' anxiety, depression, and post-traumatic stress symptoms, delivered by trained lay providers (106, 107). However, in-person group-based interventions may be less accessible in remote areas due to travel-related barriers. In such context, online formats offer a promising alternative. Group-based therapy delivered via video teleconference has been shown to yield outcomes comparable to in-person sessions, with high level of participant satisfaction (108). Moreover, online peer groups that facilitate the sharing of activities or lived experiences may further enhance engagement and expand the reach of mental health support in university populations.

The subgroup analysis did not reveal statistically significant differences across factors such as country region, theoretical orientation, delivery format, control condition, and risk of bias, suggesting that these variables alone do not fully explain the variability in effect sizes. One possible explanation for the lack of significant findings is the presence of extreme outliers, which may have disproportionately influenced the pooled results, obscuring meaningful patterns in the data. However, notably smaller effect sizes were observed in studies with higher methodological quality and in those employing control conditions other than no treatment or waitlist. Although these differences did not reach statistical significance, the consistent direction and magnitude of the effect reduction may carry clinical relevance. This pattern suggests that methodological rigor and choice of comparator condition can meaningfully influence outcome estimates. In particular, studies using active or evidence-based comparators may yield smaller between-group effects, which reflect the strength of the control rather than reduced efficacy of the intervention. Taken together, these findings highlight the importance of cautious interpretation of pooled effects, especially those derived from lower-quality studies or studies with passive control conditions. Furthermore, a sensitivity analysis was conducted by performing the subgroup analysis after excluding outliers. This analysis yielded statistically significant differences in country region, theoretical orientation, control condition, and risk of bias, indicating that outliers may have masked the effects in the original analysis (see Supplementary Material S3). The sensitivity analysis revealed that interventions conducted in the Middle East, those grounded in cognitive-behavioral theoretical orientations, studies employing waitlist control conditions, and those with a high risk of bias were associated with larger effect sizes.

Our study contributes to the growing evidence on the effectiveness of SMIs for university students in LMICs, highlighting their preventive potential in resource-limited settings. However, several limitations should be noted. First, the high risk of bias in many included studies may affect the credibility of the findings. Second, the small sample size in most studies, along with the higher proportions on first- and second-year students may limit generalizability. Third, follow-up assessments were typically short and varied considerably across studies, limiting the ability to assess long-term effects and precluding a pooled analysis of follow-up outcomes. Fourth, the presence of extreme outliers increased variability in the data, making it more difficult to detect meaningful differences in subgroup analysis and potentially obscuring sources of heterogeneity. Fifth, this review focused exclusively on studies conducted in LMICs to address a critical gap in the literature and provide context-specific evidence. While this focus adds value, it also precluded direct comparisons with studies from HICs and limited the ability to examine income level as a potential moderator. Sixth, most studies provided limited or no information on cultural adaptation, which limited our ability to examine its potential role as a moderator of intervention effectiveness. Seventh, the review included only peer reviewed studies published in English, which may have excluded relevant research published in other languages thereby limiting the comprehensiveness of the evidence base. Finally, only ten of the included RCTs were preregistered, with four providing an accessible link, which limits transparency and warrants cautious interpretation of the findings.

Transparency in this field could be strengthened if researchers in LMICs more consistently adopted preregistration of trial protocols. In the absence of preregistration, it is difficult to rule out selective reporting or post hoc analytic flexibility, both of which compromise the reliability of findings. Registering protocols on established public registries such as ClinicalTrials.gov, the International Standard Randomised Controlled Trial Number (ISRCTN) registry, or the Open Science Framework (OSF) represents a feasible minimum standard that can meaningfully enhance research credibility. In recent years, the Registered Reports (RRs) format has been increasingly recognized as a more rigorous publishing model, whereby study protocols are peer reviewed prior to data collection and granted in-principle acceptance independent of study outcomes. Evidence from recent literature indicates that the Registered Reports (RRs) format can strengthen methodological rigor by reducing publication bias, increasing the proportion of published null findings, and improving overall reporting quality (109–112). Nonetheless, the feasibility of implementing RRs in LMICs may be constrained by short funding cycles, limited infrastructure, and uneven access to journals offering this format. A pragmatic way forward may therefore be to normalize preregistration as a field-wide expectation, while fostering an environment that enables the gradual uptake of the RR model through context-appropriate adaptations aligned with local research conditions.

Despite some limitations, our findings suggest that SMIs are effective in improving stress among university students in low resources settings. This has significant implications for student mental health promotion and early prevention, as chronic stress is a known risk factor for anxiety, depression, and other mental health problems. In practice, universities could begin embedding SMIs into the academic curriculum and student support services, ensuring that mental health care is both accessible and normalized within the university environment. This may involve integrating mental health screening, structured feedback, and appropriate referrals for further support.

To maximize accessibility and minimize resource constraints, internet-based interventions present a promising alternative, offering scalable, flexible, and potentially cost-effective solutions for stress management interventions. However, digital formats- particularly those with limited or no guidance- often face challenges in sustaining user engagement. While effect size may be modest, these interventions remain valuable for non-clinical populations, especially in LMICs, where the ability to reach large groups with low intensity support can translate into meaningful public health gains.

The implementation of digital interventions, whether guided or unguided, should be grounded in ethical principles to ensure responsible use. This is especially critical in settings where users may have limited access to alternative forms of support. Ethical implementation entails ensuring informed consent, providing clear usage boundaries, offering access to referral resources, and maintaining user safety throughout the intervention process.

To address engagement challenges in digital SMIs, future implementation efforts may benefit from prioritizing minimally guided approaches that integrate human or interactive support mechanisms. Such approaches are particularly relevant in LMICs, where mental health service gaps remain substantial. Involving trained lay or peer counsellors, for example, can enhance relevance and engagement through peer-led psychoeducation, counselling, and support (113). Group-based delivery formats offer an additional layer of social interaction and cost-efficiency and can be implemented online to reach underserved student populations. These socially embedded approaches may not only increase participation but also strengthen social connectedness and resilience within university communities. The rapid development of AI technologies further expands the possibilities for enhancing digital interventions. Features such as real-time feedback, personalization, and conversational interfaces can improve user experience and adherence (101). Moreover, AI-powered tools may also support the scalability of peer- and group-based interventions by facilitating adaptive content delivery and tailored interaction at scale (101).

In addition to improving engagement, the effectiveness of SMIs may also depend on how well their content and design align with users' individual needs and contextual realities. Engagement and effectiveness are often interrelated; when interventions are perceived as relevant, acceptable, and responsive to the user's lived experience, they are more likely to produce sustained outcomes. Tailored content, culturally relevant materials, and evidence-based strategies can enhance both acceptability and therapeutic impact (114–116). In digital formats, strategies such as gamification (117), interactive features (118), and brief guidance provided by trained lay personnel (119) have been shown to support personalization and increase user engagement. In face-to-face settings, effectiveness may be promoted through interactive group discussions, peer-led sessions, and experiential learning activities that foster emotional connection and practical skill development (113, 120). A structured process of cultural adaptation, including co-design with students or localization of intervention content, may further improve contextual fit and foster meaningful engagement and outcomes (121). Finally, aligning SMIs with broader institutional mental health systems may help sustain impact by ensuring continuity of care and embedding interventions within students' academic and psychosocial environments (122, 123).

Future research directions include the following suggestions: despite the barriers LMICs face in conducting an RCT, higher quality trials are needed to provide sound evidence in this area. This may be achieved by building local researcher capacity on RCT methodologies through partnerships with established institutions for mentorship, fostering collaboration between local researchers and international experts, and increasing access to funding opportunities specifically designated for RCTs in these regions. Strengthening methodological rigor will also require greater attention to practices that enhance transparency, such as preregistration of trial protocols. Moreover, future research should examine the long-term effects of SMIs in reducing stress among university students. In addition, future meta-analyses would benefit from including studies from both LMICs and HICs, allowing for direct comparisons across economic contexts and enabling the examination of country income classification as a potential moderator. Finally, given the limited reporting in the current evidence base, more consistent documentation and integration of cultural adaptation processes is needed to better understand their contribution to intervention relevance and effectiveness.

## 5 Conclusion

SMIs are effective in reducing stress among university students in LMICs. Implementing SMIs in university setting would be a valuable step to enhance university students' well-being. To achieve this, we recommend universities in LMICs to gradually incorporating SMIs into their academic curriculum to ensure accessibility and sustainability and embedding SMIs withing student support programs. Additionally, leveraging existing resources, such as peer support networks and digital platforms, may provide scalable and cost-effective ways to expand mental health support for students in resource-limited settings. To support our conclusion, more randomized controlled trials are needed across the diverse LMIC regions represented in this meta-analysis, and future studies are expected to meet higher standards of methodological rigor to ensure more reliable and generalizable evidence.

## Data Availability

The dataset is available from the corresponding author upon request. Requests to access these datasets should be directed to dilfa.juniar@yarsi.ac.id.
