# Supplementary material for: Stress management interventions for university students in low-and middle-income countries: a systematic review and meta-analysis
Source: Front Digit Health. 2025 Sep 10;7:1603389. doi: 10.3389/fdgth.2025.1603389 (PMC12457673; doi:10.3389/fdgth.2025.1603389)
Supplement: Supplementary file 2 [file Datasheet2.docx]

***Supplementary Material***

**Supplementary Material 2. Search Query**

| **Database** | **Search query** |
| --- | --- |
| **PubMed** | (stress [tiab] OR stressed [tiab] OR distress [tiab] OR distressed [tiab] OR life stress [tiab] OR pressure [tiab] OR coping [tiab] OR coping strategies [tiab] OR behavior [tiab] OR behavioral activation [tiab] OR behavioural activation [tiab] OR coping mechanisms [All Fields]) AND (Student* [tiab] OR college [All Fields] OR university [tiab] OR university student* [tiab] OR scholar [tiab] OR higher education [tiab] OR graduate [tiab]) AND (LAMI [MeSH terms] OR LMIC [MeSH terms] OR low and middle income [All Fields] OR low- and middle-income countries [All Fields] OR low- and middle-income country [All Fields]) AND (“counseling” [All Fields] OR counsel* [All Fields] OR medication [All Fields] OR group therapy [All Fields] OR CBT [All Fields] OR PM+ [All Fields] OR behavioral therapy [All Fields] OR behavioural therapy [All Fields] OR behav* [All Fields] OR behavioral therap* [All Fields] OR cognitive therapy [All Fields] OR cognitive therp* [All Fields] OR cognition [All Fields] OR cognitive function [All Fields] OR cognitive treatment [All Fields] OR cognitive improvement [All Fields] OR behavioral treatment [All Fields] OR meta-cognitive [All Fields] OR compassion focused [All Fields] OR compassion [All Fields] OR solution [All Fields] OR solution focused [All Fields] OR self-control [All Fields] OR control [All Fields] OR mindfulness [All Fields] OR mindfulness based [All Fields] OR emotion focused [All Fields] OR emotion* [All Fields] OR training [All Fields] OR Social skills training [All Fields] OR SST[All Fields] OR internet based [All Fields] OR e-health [All Fields] OR iCBT [All Fields] OR self-help [All Fields] OR self help [All Fields]) NOT (patients [tiab] OR clinical [tiab]) |
| **Embase** | #1 ‘stress’/exp OR ‘stressed’ OR ‘distress’ OR ‘distressed’ OR ‘life stress’ OR ‘pressure’ OR ‘coping’ OR ‘coping strategies’ OR ‘behavior’ OR ‘behavioral activation’ OR ‘behavioural activation’ OR ‘coping mechanisms’  #2 Student*/exp OR ‘college student’ OR ‘university’ OR university student* OR ‘scholar’ OR ‘higher education’ OR ‘graduate’  #3 ‘LAMI’ OR ‘LMIC’/exp OR ‘low and middle income’ OR ‘low- and middle-income countries’ OR ‘low- and middle-income country’  #4 ‘counseling’ OR counsel* OR ‘medication’ OR ‘group therapy’ OR ‘CBT’ OR ‘PM+’ OR ‘behavioral therapy’ OR ‘behavioural therapy’ OR behav* OR behavioral therap* OR ‘cognitive therapy’ OR cognitive therp* OR ‘cognition’ OR ‘cognitive function’ OR ‘cognitive treatment’ OR ‘cognitive improvement’ OR ‘behavioral treatment’ OR ‘meta-cognitive’ OR ‘compassion focused’ OR ‘compassion’ OR ‘solution’ OR ‘solution focused’ OR ‘self-control’ OR ‘control’ OR ‘mindfulness’ OR ‘mindfulness based’ OR ‘emotion focused’ OR emotion* OR ‘training’ OR ‘Social skills training’ OR ‘SST’ OR ‘internet based’ OR ‘e-health’ OR ‘iCBT’ OR ‘self-help’ OR ‘self help’ |
| **PsycInfo** | “stress” OR “stressed” OR “distress” OR “distressed” OR “life stress” OR “pressure” OR “coping” OR “coping strategies” OR “behavior” OR “behavioral activation” OR “behavioural activation” OR “coping mechanisms” AND  Student* OR “college student” OR “university” OR university student* OR “scholar” OR “higher education” OR “graduate” AND  “LAMI” OR “LMIC” OR “low and middle income” OR “low- and middle-income countries” OR “low- and middle-income country” AND  “counseling” OR counsel* OR “medication” OR “group therapy” OR “CBT” OR “PM+” OR “behavioral therapy” OR “behavioural therapy” OR behav* OR behavioral therap* OR “cognitive therapy” OR cognitive therp* OR “cognition” OR “cognitive function” OR “cognitive treatment” OR “cognitive improvement” OR “behavioral treatment” OR “meta-cognitive” OR “compassion focused” OR “compassion” OR “solution” OR “solution focused” OR “self-control” OR “control” OR “mindfulness” OR “mindfulness based” OR “emotion focused” OR emotion* OR “training” OR “Social skills training” OR “SST” OR “internet based” OR “e-health” OR “iCBT” OR “self-help” OR “self help” |
