# Supplementary material for: Stress management interventions for university students in low-and middle-income countries: a systematic review and meta-analysis
Source: Front Digit Health. 2025 Sep 10;7:1603389. doi: 10.3389/fdgth.2025.1603389 (PMC12457673; doi:10.3389/fdgth.2025.1603389)
Supplement: Supplementary file 3 [file Datasheet3.docx]

***Supplementary Material***

**Supplementary Material 3.** **Subgroup analysis excluding outliers**

| Subgroup variables | Number of comparisons | Hedges' g (95% CI) | I^2^ (95% CI) | *p* |
| --- | --- | --- | --- | --- |
| Region of country | | | | |
| Middle East | 5 | -0.93[-1.22; -0.64] | 0 [0; 79.20] | <0.001 |
| Southeast Asia | 5 | -0.44 [-0.66; -0.22] | 0 [0; 79.20] |  |
| South Asia | 3 | -0.37 [-0.96; 0.21] | 25.40 [0; 92.20] |  |
| East Asia | 3 | -0.54 [-1.31; 0.24] | 47.00 [0; 84.40] |  |
| Latin America | 6 | -0.50 [-0.76; - 0.24] | 0 [0; 74.60] |  |
| Africa | 1 | -0.90 [-1.13; - 0.67] | - |  |
| Theoretical orientation | | | | |
| Mindfulness-based | 10 | -0.61 [-0.81; -0.40] | 21.2 [0; 61.30] | 0.004 |
| Psychoeducation-based | 3 | -0.55 [-1.16; 0.06] | 22.20 [0; 91.90] |  |
| Cognitive behavioral | 3 | -0.83 [-1.07; -0.01] | 0 [0; 89.60] |  |
| Mind-body | 7 | -0.37 [-1.72; -0.01] | 29.00 [0; 69.50] |  |
| Format of the intervention | | | | |
| Face to face | 17 | -0.54 [-0.71; -0.38] | 37.80 [0; 65.20] | 0.134 |
| Online | 6 | -0.72 [-0.96; -0.49] | 10.80 [0; 77.40] |  |
| Control condition | | | | |
| Waitlist | 9 | -0.79 [-0.92; -0.66] | 0 [0; 64.80] | 0.006 |
| No treatment | 11 | -0.47 [-0.67; -0.26] | 39.50 [0; 70.20] |  |
| Other | 3 | -0.46 [-1.38; 0.47] | 22.40 [0; 91.90] |  |
| Risk of Bias | | | | |
| Low & Some concerns | 14 | -0.49 [-0.64; -0.35] | 2.00 [0; 55.90] | 0.030 |
| High | 9 | -0.76 [-0.99; -0.53] | 48.30 [0; 75.90] |  |
